# Development of a Grape Cut Point Detection System Using Multi-Cameras for a Grape-Harvesting Robot

**DOI:** 10.3390/s24248035

**Published:** 2024-12-16

**Authors:** Liangliang Yang, Tomoki Noguchi, Yohei Hoshino

**Affiliations:** 1 Laboratory of Bio-Mechatronics, Faculty of Engineering, Kitami Institute of Technology, Koentyo 165, Kitami Shi 090-8507, Hokkaido, Japan; tomoki.noguchi@yaskawa.co.jp (T.N.); hoshinoy@mail.kitami-it.ac.jp (Y.H.); 2 YASUKAWA Electric Corporation, 2-1 Kurosakishiroishi, Yahatanishi-ku, Kitakyushu 806-0004, Japan

**Keywords:** grape-harvesting robot, multi-camera system, you only look once (YOLO) based grapes detection, stem detection using semantic segmentation

## Abstract

Harvesting grapes requires a large amount of manual labor. To reduce the labor force for the harvesting job, in this study, we developed a robot harvester for the vine grapes. In this paper, we proposed an algorithm that using multi-cameras, as well as artificial intelligence (AI) object detection methods, to detect the thin stem and decide the cut point. The camera system was constructed by two cameras that include multi-lenses. One camera is mounted at the base of the robot and named the “base camera”; the other camera is mounted at the robot hand and named the “hand camera” to recognize grapes and estimate the stem position. At the first step, the grapes are detected by using a You Only Look Once (YOLO) method, while the stems of the grapes are detected at the second step using a pixel-level semantic segmentation method. Field experiments were conducted at an outdoor grapes field. The experiment results show that the proposed algorithm and the camera system can successfully detect out the cut point, and the correct detection rate is around 98% and 93% in the indoor and outdoor conditions, respectively. The detection system was integrated to a grape-harvesting robot in the experiment, and the experiment results show the system can successfully harvest the grapes in the outdoor conditions.

## 1. Introduction

Grapes are the fourth most produced fruit all over the world, following bananas, watermelons, and apples. The current grape-harvesting process mainly uses manual labor. The harvesting job requires a significant amount of time and labor. Research has shown that automating and mechanizing the harvesting process could improve efficiency [1]; in particular, utilizing harvesting robots can reduce the labor required for crop management when maintaining the same quality [2]. In the research field of fruits and vegetables harvesting robots, an efficient cucumber-harvesting robot system was designed [3]. In addition, an automated harvesting of individual cherry tomatoes was developed and tested in the greenhouse environment [4]. For harvesting irregularly oriented citrus stems, an end effector for citrus-harvesting robots was designed [5]. This research was mainly conducted to finish the hardware of a harvesting robot instead of focusing on how to detect the target, although the detection is the first step of a harvesting robot. Therefore, it is necessary to research how to detect the fruit targets and the cut point of the fruits.

In recent years, there have been some famous models in the field of artificial intelligence (AI) utilizing deep neural networks (DNN) for object detection that can be used for the detection of fruits and vegetables for automatically harvesting. You Only Look Once (YOLO) [6], Faster Regions with Convolutional Neural Networks (RCNNs) [7], and Single-Shot MultiBox Detector (SSD) [8] were initially introduced for multi-class recognition. While the YOLO method demonstrated a well-balanced detection model compared to Faster RCNN and SSD, it was observed that YOLO’s recall is relatively lower. To address this limitation, YOLOv2 [9] was introduced in 2016, incorporating normalization and concatenation layers into each CNN. In 2018, YOLOv3 [10] further enhanced the model by integrating scale-wise predictions, enabling object detection at three different scales to accommodate various object sizes within an image. Subsequent versions such as YOLOv4 [11], YOLOv5, and YOLOX [12] were constructed based on the YOLOv3 network. Later successors, YOLOv7 [13] succeeding YOLOv4, and YOLOv8 succeeding YOLOv5, were developed, incorporating various improvements in network structure, data augmentation, and anchors.

The AI-based object detection algorithm was introduced for the accurate identification of fruits and vegetables in recent years. For example, a mobile harvesting module using multiple arms in a greenhouse adopted a CNN model—YOLOX [14]. In addition, a grape detection method based on improved YOLOv4-Tiny was used for a grape-picking robot [15]. An end-to-end lightweight counting pipeline was developed for the real-time tracking and counting of grape clusters in field conditions. A soft non-maximum suppression is introduced in the prediction stage to improve the detection performance for clusters with overlapping grapes [16]. In the vineyards, the grape clusters and grape stems are often heavily obscured by leaves, branches and clusters, making it difficult to distinguish them accurately. A DualSeg method was proposed by leveraging the advantages of CNN at local processing and transformer at global interaction [17]. At the same time, there is research still being conducted on fruit detection without AI, such as normalizing surface illumination and implementing K-means clustering for fruit image segmentation [18], and research on apple branch segmentation utilizing contrast-limited adaptive histogram equalization (CLAHE) for local contrast adjustment and histogram equalization [19]. These methods show that the traditional image processing methods still have the space to be utilized in the harvesting robot field.

Researchers developed a robust accurate method for the detection and localization of the peduncle of table grapes with direct implementation in automatic grape harvesting with robots [20]. A multi-joint robotic arm and a 3D camera (Realsense) attached to the end effector was utilized for the development of a harvesting robot [21]. This approach determines a hand–eye path for depth perception, defining three key points—the far point, the near point, and the picking point. Data acquired at these locations are synthesized using an algorithm to estimate an overall harvesting point. This method can only be used at an idea condition when the cut point is invariably defined directly above the central line of the grape cluster.

In addition to the object detection of fruits, it is important to obtain the 3D coordinates of the detected fruits for harvesting, which can be achieved by using LiDAR [22,23,24] or a binocular stereo (BS) camera [25,26]. A method for acquiring spatial information from grape clusters has been presented based on binocular stereo vision [27]. The Time of Flight (ToF) method [28,29] calculates the round-trip distance between the sensor and the object, which can provide a higher accuracy than the BS camera, while the price of the ToF 3D camera is much higher than that of the BS camera.

From previous studies, it can be seen that the thin stem of grapes has not been researched, which is important for the grape-harvesting robot. Therefore, we are focused on the development of an algorithm that can correctly detect the thin stem and decide the cut point using multi-cameras in this study, because one color camera cannot detect the color and 3D information. This paper features a multi-camera system that includes a BS camera mounted at the base of the harvesting robot in order to roughly detect the position of the grape clusters and a ToF 3D camera mounted at the robot hand to precisely detect the position of the grape clusters as well as the cut point. This paper is organized as follows: Section 2 shows the materials and methods of this paper, which include the detail of the hardware system as well as the detail of the detection algorithm; Section 3 shows the experiment results in the indoor and outdoor conditions and discussion of the results; Section 4 concludes this paper.

## 2. Materials and Methods

### 2.1. The Grape-Harvesting Robot System

**Figure 1:**
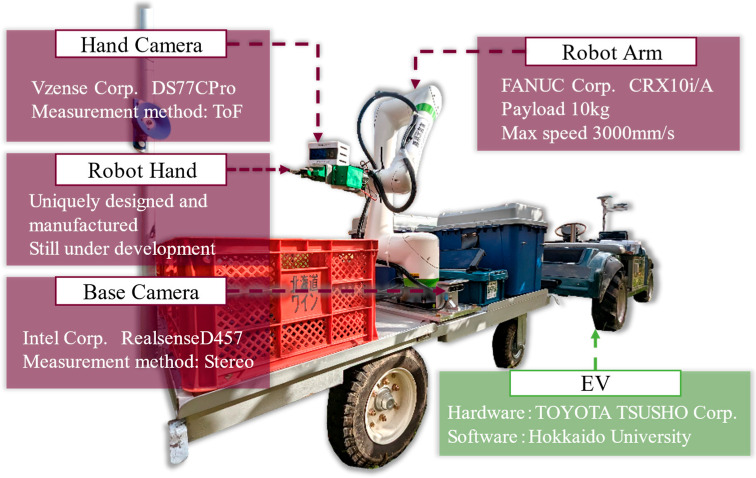
The configuration of the harvesting robot.

The configuration of the harvesting robot is shown in Figure 1. The harvesting robot consists of a robot arm, a base camera installed on the base, a robot hand and a hand camera. Containers for harvesting grape bunches are loaded in the space of the cart. The harvesting module is towed by an EV-type vehicle. A 1500 W inverter is connected to a 12V battery, and the AC100V output of the inverter powers the robot arm (CRX10i/A, Fanuc Corp., Yamanashi, Japan) and the other devices.

**Figure 2:**
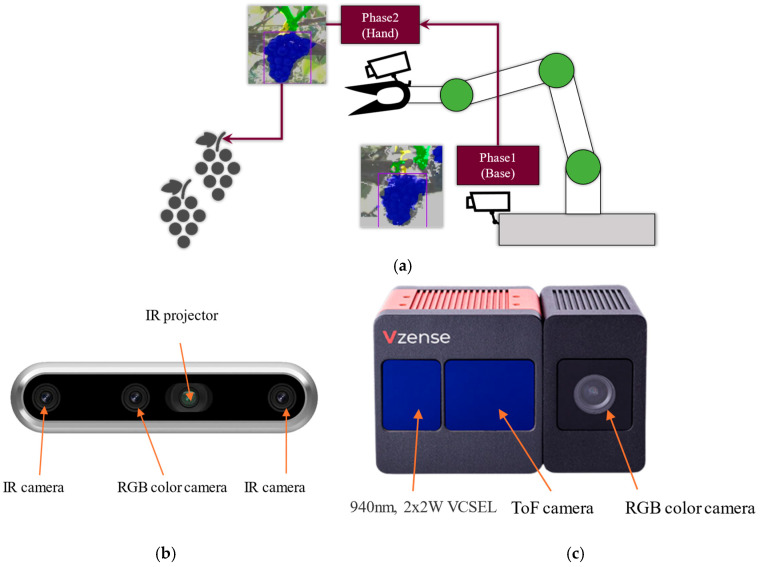
Multi-camera system of the grape-harvesting robot: (**a**) camera system; (**b**) base camera; (**c**) hand camera.

In order to detect the cut point of grapes for harvesting in the vineyards, two cameras were utilized in this study, as shown in Figure 2. One is a base camera that is used to roughly detect where the grapes cluster is; the other is used to precisely detect the position of the grapes cluster, branch, and finally decide the position of the cut point. For the base camera, we used a stereo 3D camera (RealSense D457, Intel Corporation, Santa Clara, CA, USA) that can obtain depth information without the thin objects. For the hand camera, we used a ToF-type 3D camera (DS77C pro, Vzense Technology, Qingdao, China) that can acquire more detailed depth information than the stereo type. The specifications of the two cameras are shown in Table 1.

### 2.2. Grape Cluster and Cut Point Detection Method

#### 2.2.1. The Process Steps of Cut Point Detection and Harvesting Robot

**Figure 3:**
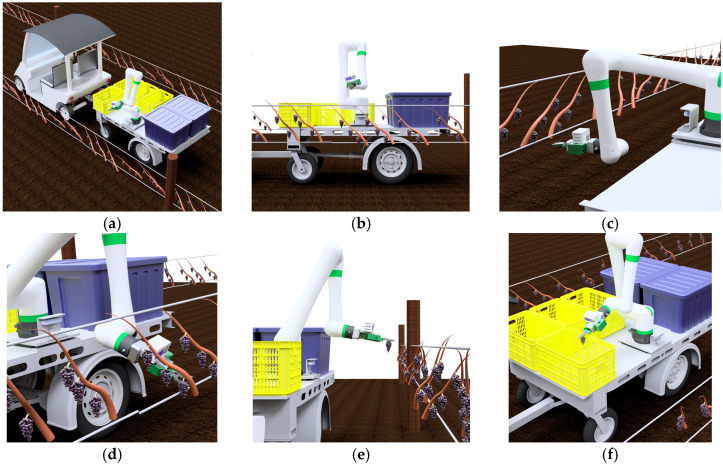
Harvesting process with the grape-harvesting robot: (**a**) moving through the vineyard; (**b**) grape and cut point detection; (**c**) move to cut point; (**d**) cut at the cut point and hold the stem; (**e**) take away from the vine; (**f**) carry to container.

The harvesting process of the grape-harvesting robot is shown in Figure 3. At first, the robot is going through along the rows of the grapevines automatically, as shown in Figure 3a, and image data of grapevines are acquired while driving. When a grape cluster is detected, the system sends a stop command to the vehicle to let the vehicle stop. Second, as shown in Figure 3b, cut points within the field are detected using AI recognition; this paper is mainly focused on this step.

And at the third, the robot arm moved to the estimated grape coordinates as shown in Figure 3c. Then, the robot hand as the end effector cuts and grips the grapes as shown in Figure 3d. The robot arm takes away from the grapevines slowly as shown in Figure 3e. Finally, the harvested grapes are transported to a container in Figure 3f. The harvesting operation is continued until finishing the harvesting within the field of view of the camera and within the harvestable range. The harvestable range refers to the maneuverable space of the robot arm that avoids interference with other machines.

**Figure 4:**
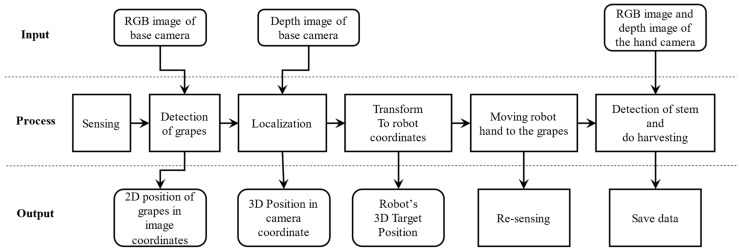
The process steps of cut point detection and harvesting robot.

The software’s main structure for the cut point detection and harvesting robot are shown in Figure 4. Moreover, the data input and output data of each step are shown besides the process steps. The process steps of the recognition system include sensing, detection, localization, transform, moving and re-sensing, which were designed to detect and locate the grapes as well as the cut point. Then, it produces the cut point position and sends the position result to the robot hardware to perform the harvesting job.

At the sensing step, the base camera takes the red, green and blue (RGB) color image data and stereo image data at the same time. The RGB color image is used to detect the grapes in order to determine the grapes to be harvested, because the cut point is difficult to be seen around the grapes due to obstacles such as the branches and stems. The grapes’ 3D position is calculated from the stereo image data by extracting the grapes area from the detection step. Then, the 3D position will be transformed to the robot coordinates, and the robot hand is moved to the front around 0.5 m before the detected grapes in order to see the grapes at a near distance. In addition, the hand camera will perform the sensing job after the robot hand has finished its movement. The RGB color image and 3D point cloud data will be grabbed at the same time from the hand camera, which is a ToF camera. The stem detection job will be completed here to produce the cut point position in the robot arm coordinates. The produced cut point position is sent to the robot to finish the cutting and storage jobs. After completing a series of harvesting operations, the robot performs the harvesting operation again until finishing the grapes harvesting within the harvestable range.

#### 2.2.2. Data Collection for Training the AI Detection Model

**Figure 5:**
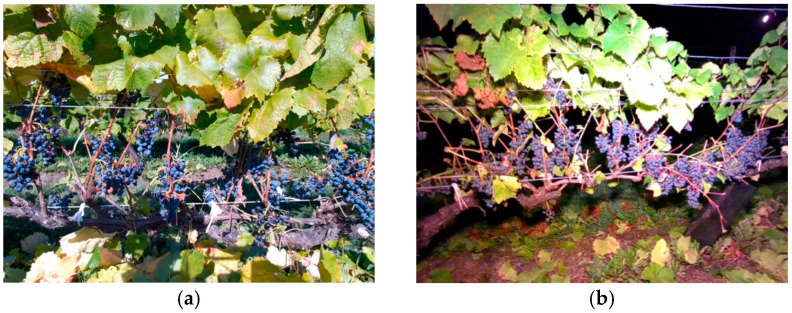
Data sample of the collected data using RGB image: (**a**) daytime image data; (**b**) nighttime image data under light.

In order to detect the grapes and the cut point, we collected image data in a real vineyard of Tsurunuma Winery located at Urausu-cho, Hokkaido Japan during the harvesting season from 2022 to 2023. RGB images and IR images were captured during daytime and nighttime. The RGB images were only used as training data, because the IR images lost the color information of the grapes. Data were acquired from a distance of approximately 1 m from the vines using the base camera and hand camera. The difference between the daytime and nighttime data is shown in Figure 5. The back row is visible in the daytime image, while only the front row is visible in the night image.

#### 2.2.3. Detection of the Grape Cluster Using the Base Camera

As shown in Figure 4, the grape cluster is detected at first using YOLO models including YOLOv3, YOLOv5, YOLOv7, and YOLOv8 constructed in Python 3.12.5 using the PyTorch library. The training and test datasets contained 340 and 70 images, respectively. The 340 images are divided in the ratio of 70% and 30% for training and validation, respectively. The accuracy of the object detector for a specific dataset is evaluated using the Intersection over Union (IOU) method. IOU requires two types of data: the ground truth data (TrueBox) and the predicted bounding boxes from the model (PredictBox). When expressed as TrueBox and PredictBox, IOU is represented by Equation (1). In this way, IoU reflects the ratio of the overlapping region between TrueBox and PredictBox.

(1) $$IoU=\frac{Area\;of\;Intersection\;of\;two\;boxes}{Area\;of\;Union\;of\;two\;boxes}$$

The implementation method is outlined below. The coordinates of the top-left corner of TrueBox are ($x_{t1},y_{t1}$) and the bottom-right corner are ($x_{t2},$ $y_{t2}$). Similarly, for PredictBox, the coordinates of the top-left corner are ($x_{p1},y_{p1}$) and the bottom-right corner are ($x_{p2},y_{p2}$). To calculate the top-left corner of the intersection, compare the top-left corners of each box. $x_{inter1}$ is determined by whether the top-left corner of the box is to the right, and $y_{inter1}$ similarly considers whether the top-left corner of the box is lower than the others.

(2) $$x_{inter1}=\max\left(x_{t1},x_{p1}\right),\;y_{inter1}=\max\left(y_{t1},y_{p1}\right)$$

To calculate the bottom-right corner of the intersection, compare the bottom-right corners of each box. $x_{inter2}$ is determined by which box has its bottom-right corner more to the left. Similarly, $y_{inter2}$ compares the heights of the bottom-right corners of the two boxes on the image.

(3) $$x_{inter2}=\max\left(x_{t2},x_{p2}\right),\;y_{inter2}=\max\left(y_{t2},y_{p2}\right)$$

When the boxes overlap completely or have the same value at specific coordinates, the comparison of minimum and maximum values simply becomes the value itself. Now that we have the coordinates of the intersection, the area of the intersection is simply the area of the formed rectangle. (Even if the order of the boxes changes, take the modulus of width and height to ensure positive values for both the width and height.) If $x_{inter1}>x_{inter2}$, the width will be a positive value.

(4) $${Width}_{inter}=\left(x_{inter2}-x_{inter1}\right)$$

(5) $${Height}_{inter}=\left(y_{inter2}-y_{inter1}\right)$$

(6) $$Area\;of\;Intersection\;of\;two\;boxes={Width}_{inter}*{Height}_{inter}$$

(7) $$\begin{array}{c} Area\;of\;Union\;of\;two\;boxes=\\ \left({Width}_{1}*{Height}_{1})+{(Width}_{2}*{Height}_{2})-{(Width}_{inter}*{Height}_{inter}\right) \end{array}$$

where,

$${Width}_{1}={(x}_{p1}-x_{t1}),\;\;{Height}_{1}=\left(y_{p1}-y_{t1}\right)$$

$${Width}_{2}={(x}_{p2}-x_{t2}),{\;\;Height}_{2}=\left(y_{p2}-y_{t2}\right)$$

The IOU between two boxes ranges from 0 to 1. If there are two non-overlapping boxes, the area of their intersection is 0, and IOU is also 0. In the case of two boxes completely overlapping, the area of the intersection equals the area of their union, resulting in an IOU of 1. We computed the IoU for each image in the test data and measured the inference time. The average values for each of the 70 images were calculated to obtain the mIoU and average inference time. Table 2 presents the results for the IOU and inference time of YOLOv5, YOLOv7, YOLOv6 and YOLOv8.

**Figure 6:**
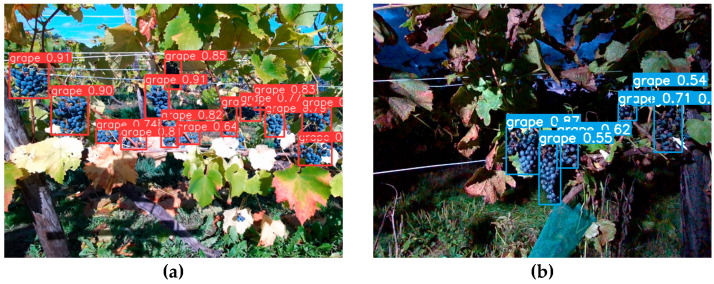
Detection result of grapes using YOLOv8s: (**a**) daytime; (**b**) nighttime.

Networks with shallower layers tend to be more practical even among models of the same type. Particularly, YOLOv8s, YOLOv7_tiny, and YOLOv5s exhibit superior performance in terms of IOU and inference speed compared to other models. Based on the above inference comparison results, we incorporate the model YOLOv8s into the inference program on the control PC for use as inference AI models. The prediction results for the model YOLOv8s are shown in Figure 6.

#### 2.2.4. Cut Point Detection and 3D Position Estimation from Hand Camera

To address the challenge of harvesting wine grapes, where harvesting is performed at a bunch of grapes, it is crucial to recognize the grape cut point similar to manual harvesting. Pixel-wise object category identification using a semantic segmentation model is used for cutting point recognition.

Initially, a semantic segmentation model with the entire image as input was used for AI recognition, covering the detection of harvestable grapes and the approach to cut points. However, cut points, compared to other elements of the grapevine, have a smaller recognition area and are prone to occlusion. Consequently, using the entire image as input resulted in a lack of detailed recognition results for the grapevine. Additionally, unnecessary data such as the ground and sky outside the harvestable range were included in the input, leading to reduced recognition accuracy. Classifying individual elements like grape clusters and result branches became challenging, and instances of cut point recognition in regions without grape clusters were observed, resulting in frequent misrecognition and undetected cut points.

**Figure 7:**
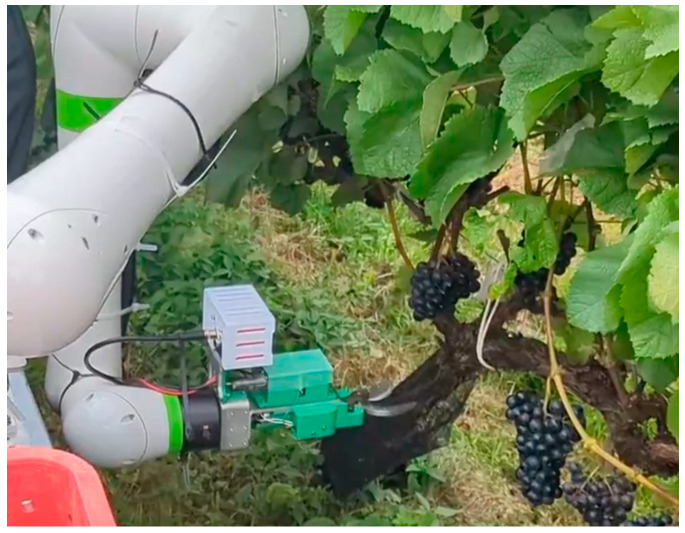
Recognition condition of hand camera.

In this study, a ToF 3D camera (hand camera) on the robot hand repeated the detection of grape bunches, recognition of cut points, and estimates the coordinate values to determine the target coordinates for harvesting with higher accuracy. The recognition condition is illustrated in Figure 7, featuring the grape bunch coordinate estimation system using the hand camera.

**Figure 8:**
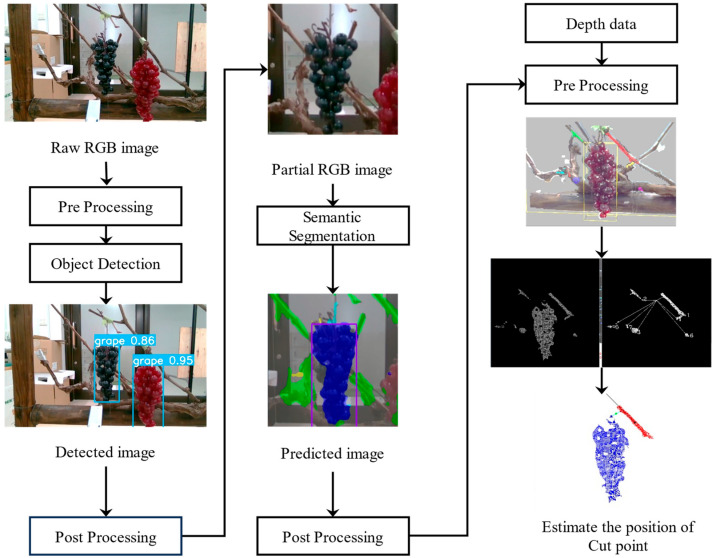
Cut point detection and its coordinate estimation system of the hand camera.

As shown in Figure 8, a multi-step recognition algorithm utilizes two CNN models for object detection and segmentation in the hand camera, respectively. The advantage of separating the object detection and segmentation models is that multiple models can be conducted without affecting the recognition accuracy of each model. The robot hand is moved to the target coordinates estimated in each step using the camera-to-robot coordinate transformation described in the following.

**Figure 9:**
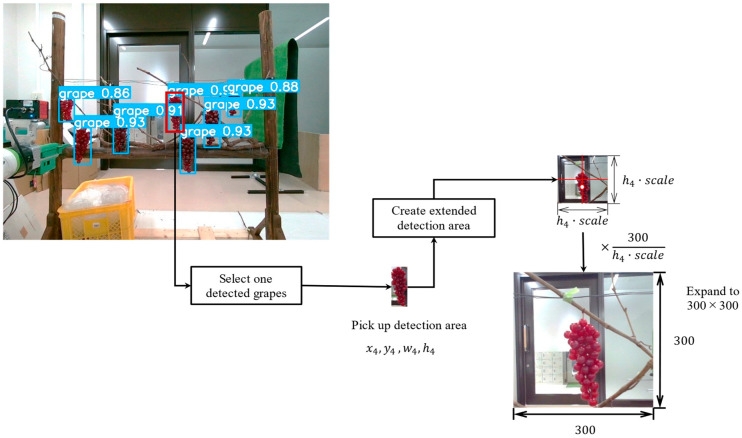
Extraction of the grapes ROI in the image.

At the first step, the entire vine image is used as the input image. The object detection model is employed to detect grapes. The detected grape cluster coordinates are estimated by extracting the corresponding region of 3D data obtained from the depth image of the 3D camera. However, only the grape cluster region is considered from the recognition results in this step. To enable the recognition of essential components, a square image of a constant size (300 × 300 pixels) is created based on the detected grapes, as shown in Figure 9. In this study, ${Cx}_{detect}$ and ${Cy}_{detect}$ represent the center coordinates of the detected grapes, and $W_{detect}$, $H_{detect}$, and scale denote the width, height, and expansion ratio of the grape cluster detection region, respectively. The center coordinates and region of the semantic partial extraction image are defined by Equations (8) to (10). In 2D images, grapes are often vertically elongated rectangles. Figure 8 assumes $W_{detect}\geq$ $H_{detect}$; however, if this is not the case, $H_{detect}$ is replaced with $W_{detect}$. In the field experiments of this study, the scale was set to 1.3.

(8) $$u_{PEI}={Cx}_{detect}-\left(\frac{H_{detect}\cdot scale}{2}\right)$$

(9) $$v_{PEI}={Cy}_{detect}-H_{detect}\cdot scale$$

(10) $$w_{PEI}=h_{PEI}=H_{detect}\cdot scale$$

For the recognition of branch and cut points, the center ${Cy}_{PEI}$ of the moved region of interest image can be expressed as shown in Equation (11).

(11) $${Cy}_{PEI}={Cy}_{detect}+\left(\frac{H_{detect}\cdot \left(1-scale\right)}{2}\right)$$

The extracted ROI image is enlarged to a fixed size of 300 × 300 pixels. This specific size is derived from the image dimensions of grape clusters captured during close-range photography using the hand camera.

**Figure 10:**
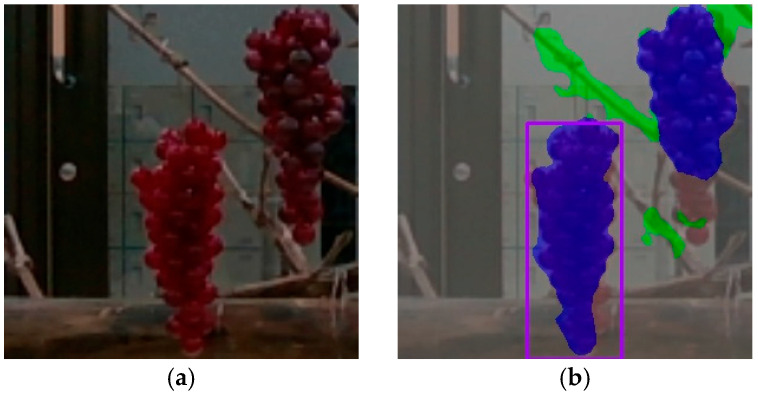
Extraction of grapes to be harvested based on grape detection results: (**a**) raw data; (**b**) recognition result. The purple square is the grapes that will be harvested.

However, as shown in Figure 10, there is a possibility of multiple grape clusters being present from the semantic segmentation model’s recognition results. Therefore, by utilizing the coordinates of the bounding box obtained from the object detection results, areas outside the bounding box are considered as other grape clusters, while the areas inside the bounding box are identified as the target grape cluster for harvesting. The detection results of the object detection model are represented by purple rectangles in this figure.

The coordinates of the harvestable grape cluster region are obtained from the 3D camera’s depth image. The depth image of the 3D camera, as mentioned in the previous section, is an image obtained by matching the depth image to the color image. To interpolate missing depth information in the depth image, a median filter is applied. The median filter is adept at removing salt-and-pepper-like noise. It orders the pixel values in the processing pixel and its neighborhood in ascending order, and the output is the median value when sorted. In this study, a 3 × 3 kernel is used.

**Figure 11:**
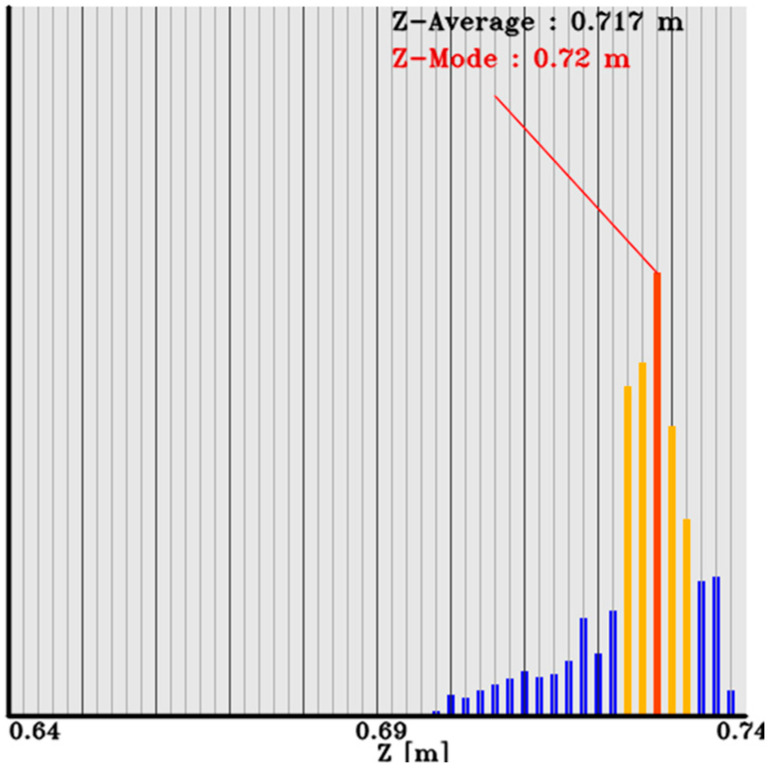
Histogram of Z-directional coordinate values of grapes. The blue bars show the raw data; the orange bars show the selected region to compute the mode value and average value; the red bar shows the mode value.

To estimate the most grapes-like coordinate value from multiple depths (Z-coordinate values), as shown in Figure 11, the Z-coordinate values are histogrammed in 2 mm increments. After histogramming, the mode value is used to define the effective coordinate value range by considering the elements within ±5 cm of the mode value. The effective coordinate values are then determined, and the Z-coordinate value of the harvestable grapes is set as the average value within a range of 15% of the effective coordinate values on both sides of the mode value. In case of multiple mode values, the smaller coordinate value side (closer to the robot base) is chosen to avoid collisions with grapes or branches. The XY coordinates of pixel positions with Z-coordinate values within the effective coordinate value range, which extend 15% of the effective coordinate value count on both sides of the mode value used in the estimation of the harvestable grape’s Z-coordinate value, were obtained. These coordinates were then used along with the camera’s intrinsic parameters to calculate the XY values.

To stabilize the estimated coordinates, a low-pass filter (LPF) was applied to the values. The continuous-time transfer function s of the LPF in the continuous-time domain was replaced by the bilinear transform, resulting in Equation (12), and a discrete-time transfer function was created.

(12) $$s=\frac{2\left(1-z^{-1}\right)}{∆T\left(1+z^{-1}\right)}$$

Here, T represents the sampling period (the control cycle of the program), and τ is the time constant. By substituting t−1 with $Z^{-1}$, the discrete-time transfer function is expressed as Equation (13).

(13) $$\frac{1}{1+\tau s}=\frac{1}{1+\tau \left(\frac{2(1-z^{-1})}{∆T(1+z^{-1})}\right)}\;=\frac{1+z^{-1}}{∆T+∆TZ^{-1}+2\tau (1-z^{-1})}\;=\frac{1+z^{-1}}{1+Z^{-1}+\frac{2\tau }{∆T}-\frac{2\tau }{∆T}z^{-1}}\;=\frac{1+z^{-1}}{1+\frac{2\tau }{∆T}+\left(1-\frac{2\tau }{∆T}\right)z^{-1}}$$

$x[k\pm n]=z^{\pm n}\cdot x\left[k\right]$; the relationship between out and in can be expressed as in Equation (17).

(14) $$out\left[k\right]=\frac{1+z^{-1}}{1+\frac{2\tau }{∆T}+\left(1-\frac{2\tau }{∆T}\right)z^{-1}}in\left[k\right]$$

(15) $$out\left[k\right]\left(1+\frac{2\tau }{∆T}\right)+out\left[k\right]\cdot z^{-1}\left(1-\frac{2\tau }{∆T}\right)=in\left[k\right]+z^{-1}\cdot in\left[k\right]$$

(16) $$out\left[k\right]\left(\frac{∆T+2\tau }{∆T}\right)=in\left[k\right]+in\left[k-1\right]-out\left[k-1\right]\left(\frac{∆T-2\tau }{∆T}\right)$$

(17) $$out\left[k\right]=\left(\frac{∆T+2\tau }{∆T}\right)\left(in\left[k\right]+in\left[k-1\right]\right)-\left(\frac{∆T-2\tau }{∆T+2\tau }\right)out\left[k-1\right]$$

**Figure 12:**
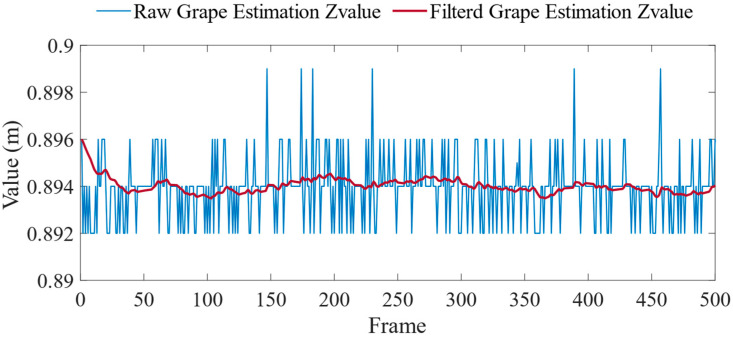
Histogram of Z-directional coordinate values of grape bunches.

The output of the discrete-time LPF, denoted as $out\left[k\right]$, can be calculated based on the previous output value, input value, and the previous input value. The LPF was implemented in the program, and filtering was applied to the estimated Z-coordinate value, X-coordinate value, and Y-coordinate value of the grape bunch. The control cycle was set to 0.033 s (30 Hz), and the time constant was set to 0.066. Figure 12 illustrates the filtering result of the Z-coordinate value of grape bunches.

The computed average values of the XY coordinates were considered as the harvested grape’s X-coordinate value and Y-coordinate value, respectively.

The process of obtaining the coordinates of the harvest target grape bunch and cut points from the 3D camera depth image is applied to both grape bunch and cut point coordinates. Since cut points cannot be distinguished by object detection like grape bunches, individual regions are detected based on their contours. A filter is constructed to exclude regions where the center of gravity is below the center of the grape bunch region and regions where the angle between the line connecting the top point of the grape bunch region and the center of gravity of the cut point region is greater than 45 degrees.

When cut points cannot be recognized or are misrecognized by grapes, we proposed two methods—to estimate cut points from only grapes and to estimate cut points from grape bunches and branches—and used the method with the higher confidence level.

The first method estimates the cut point from grapes directly, which is 15 mm above the Y-coordinate value of the top point of the harvest grape bunch. The X-coordinate value of the cut point is the harvest grape bunch’s X-coordinate value. In addition, the Z-coordinate value of the harvest grape plus 45 mm is set as the cut point Z-coordinate value.

**Figure 13:**
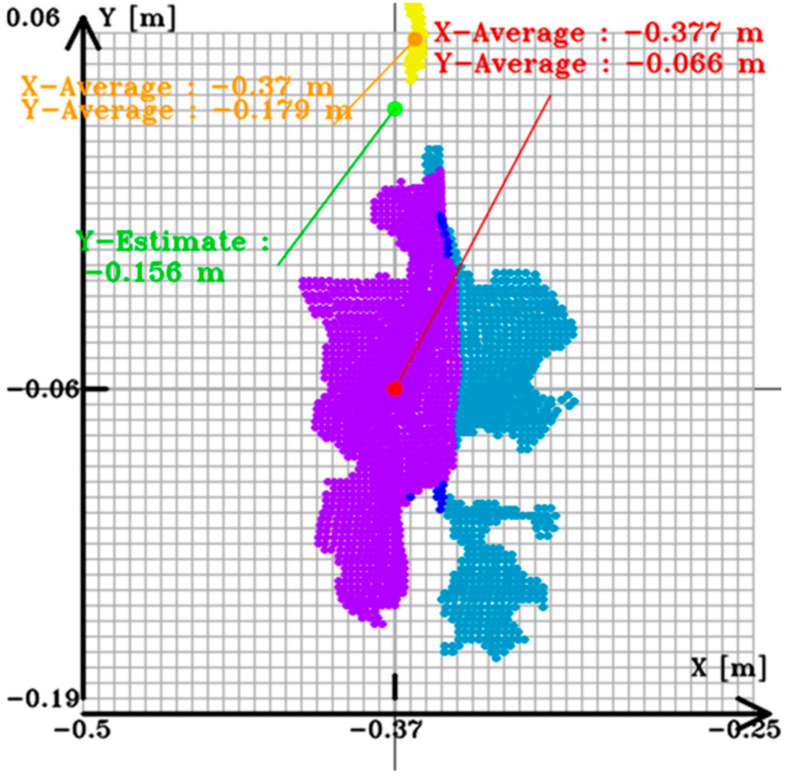
Detected grapes and cut point of the hand camera. The purple region is the selected point cloud used for calculation. The blue region is the point cloud that is a little far away from the purple region.

The distribution of grape bunch and cut point XY coordinates, centered around the harvest grape XY coordinate values, is illustrated in Figure 13. In this figure, the purple region is the grape bunch region that is used for calculation, while the blue region is not used for the distance and is far away from the purple region. The yellow point is the point in the stem, and the green point is the estimated cut point.

The second estimation method estimates the cut point from the grape bunch and branch together. After segmentation recognition, Z-coordinate values are obtained for each region in the post-processed label image. The obtained coordinates’ XY-direction values are then acquired.

**Figure 14:**
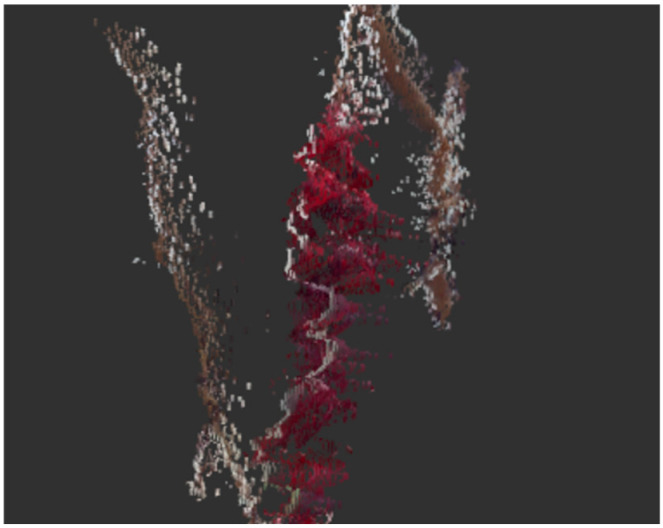
Point cloud noise around the stem (view of grapevines from the side).

Due to the elongated structure of the branches, measurement noise, as illustrated in the Figure 14, occurs frequently. To address such measurement errors, statistical analysis is conducted for the neighborhood of each point, and points that do not meet certain criteria are discarded. This approach helps mitigate the impact of measurement inaccuracies in the estimation process.

To segment the filtered point cloud into individual branches, clustering is employed. Clustering techniques enable the subdivision of the point cloud P into smaller segments, significantly reducing the overall processing time for P. In a Euclidean sense, a straightforward data clustering approach can be implemented using an octree structure rather than 3D grid subdivision with fixed-width boxes. This specialized representation can be constructed very rapidly and is particularly useful when a volume representation of occupied space is necessary or when the data within each 3D box can be approximated with different structures.

**Figure 15:**
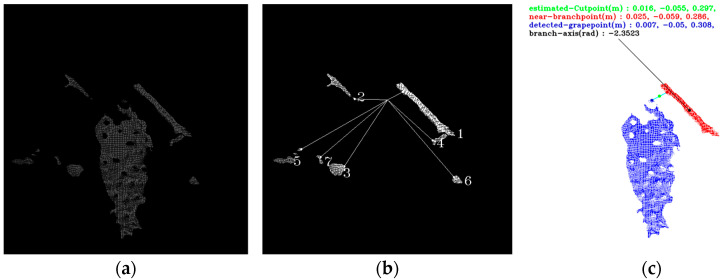
Cut point estimation from the point cloud of target grape and near-branch: (**a**) point cloud of target grapes and branches; (**b**) nearest neighbor search results; and the number shows the neighbor objects order; (**c**) estimation of the cut point.

To determine the branches supporting the harvested grape clusters, as shown in Figure 15, a nearest neighbor search is conducted between the clustered point clouds and the topmost point of the harvested grapes. Branch point clouds are excluded if their nearest neighbors have a lower Y-coordinate than the topmost point of the target grapes and if the slope of the line connecting the nearest neighbor within each branch point cloud to the topmost point of the target grapes exceeds 45 degrees.

Among the remaining branch point clouds, the one with the shortest length of the line connecting its nearest neighbor to the topmost point of the target grapes is identified as the “near-branch” and considered as the branch associated with the target grapes cluster. The figure illustrates clustered branch point clouds, nearest neighbor search results, and the estimated cut points based on the near-branch and target grapes point cloud.

The midpoint of the predicted branch is estimated as the cut point. Three types of cut point coordinates are compared: coordinates directly obtained from the recognition region (AI-Cut point), coordinates predicted only for grape clusters (Grape-Cut point), and coordinates predicted based on both the result branches and grape clusters (G&B-Cut point). The most reliable coordinates are selected by comparing these three types of coordinates. The selection process is performed over 8 frames rather than a single frame, covering the entire phase from object detection to coordinate estimation, to ensure stability. The priority order is AI-Cut point, G&B-Cut point, Grape-Cut point.

## 3. Experiment Results and Discussion

### 3.1. Indoor Experiment Results and Discussion

**Figure 16:**
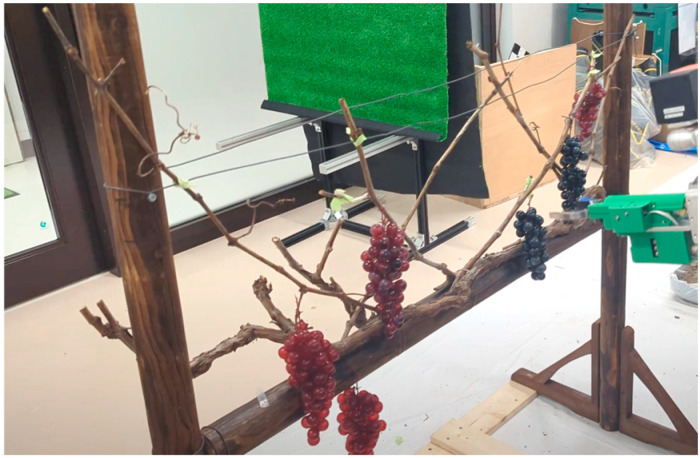
The experiment at indoor condition using replica grapevine.

In this study, we fabricated a simulated grapevine resembling a grapevine and conducted indoor harvesting experiments as shown in Figure 16. Grapes were arranged between horizontal poles with an internal width of 1000 mm, which were positioned between 600 mm and 1000 mm above the ground. The branches used were pruned and collected from the branches of the experimental field. The fabricated simulated grape vine is shown in Figure 16. Grapes used for the simulated grapevine were based on food samples, with dimensions approximately 80 mm × 80 mm × 200 mm, and a weight of around 80 g. The replica grapes were constructed by using a resin material for the cluster part and real grape stems collected for the axis. These components were combined to create the replica grapes. The grape clusters were attached to a mock grapevine using tape, and the stem length was adjusted during the experiments. To closely simulate actual field experiment conditions, the distance between the grapevine and the harvesting robot was set to approximately 700 mm, and the installation position of the harvesting robot was 700 mm above the ground.

We performed harvesting experiments with five different lengths of stem—20 mm, 30 mm, 40 mm, 50 mm, and 60 mm—to represent the entry range of the current robot. The results of 20 harvesting approaches for each length are presented in Table 3. The success rates were 70% for 20 mm, 75% for 30 mm, 90% for 40 mm, 95% for 50 mm, and 100% for 60 mm, resulting in an overall success rate of 90%. Grape clusters with spike axis lengths of 40 mm or more were almost entirely harvestable.

In the indoor experiments, it was challenging to replicate the intricate variations and angles present in actual grape trees. Consequently, prominent factors contributing to failures include the misalignment of coordinate values due to the misrecognition of cut point coordinates. The result of the second-stage semantic segmentation is very important to detect the thin stem; we hoped that it will be substantially improved by increasing the training data and enhancing recognition accuracy.

### 3.2. Outdoor Field Experiment Results and Discussion

**Figure 17:**
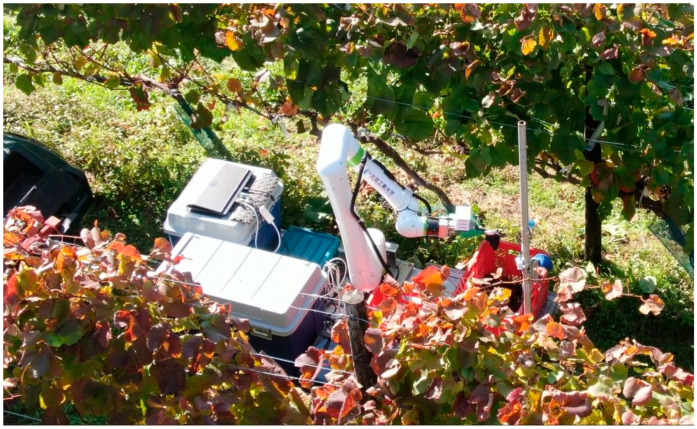
Outdoor field experiment condition.

We conducted grape-harvesting experiments in the grape fields of Tsurunuma Winery, which is a limited company in Hokkaido, Japan. The experimental setup during harvesting is illustrated in Figure 17. A grape-harvesting robot, comprising a collaborative robot arm, harvesting robot hand, base camera, control unit, battery, and other components, was mounted on a towing cart to form the harvesting module. After harvesting, two containers were loaded with harvested grapes and towed by an electric vehicle (EV). During the harvesting process, the EV was towed to the location of grape clusters, and if grape clusters were present, the EV came to a halt. After confirming the stop, the grape clusters were harvested. Therefore, harvesting operations were not performed on clusters that were clearly impossible to harvest.

The grapevines in the experimental field are trained in VSP. The experiments were conducted on a total of 72 grapevines. The experimental dates were during the harvesting season and under sunny weather conditions. The robot approaches the grape cluster detected by the base camera, adjusts the orientation of the robot hand based on the coordinates recognized by the hand camera, and then enters the cutting and gripping operation. After completing the cutting and gripping motion, the robot temporarily moves away from the grapevine and transports the grape cluster to the container. The results of the cut point detection are shown in Table 4.

## 4. Conclusions

This study aimed to develop a wine grape-harvesting robot to reduce labor requirements, focusing on the algorithm for detecting the grape stem cut point. The algorithm uses AI recognition technology based on a multi-camera system comprising a base camera (with one RGB and two IR cameras for cluster detection and distance estimation) and a hand camera (with an RGB and ToF 3D camera for recognizing grapes, stems, and cut points). The algorithm first employs semantic segmentation to estimate cut point positions from point clouds. If obstacles such as leaves or branches obstruct the view, a secondary method uses point clouds to estimate the cut point based on the closest grape branch. Experiments in controlled indoor and natural outdoor environments demonstrated 98% accuracy in detecting cut points for replica grapes with stem lengths of 40–60 mm and 93% accuracy for real grapes in outdoor conditions. These results indicate the algorithm effectively recognizes and locates cut points, which have been integrated into the robotic harvester for cutting and gripping. Current experiments were conducted with purple grapes, and future tests will address different grape colors, such as green and white, in various fields.

## Figures and Tables

**Table 1:** Specifications of the cameras used in this study.

Item	Base Camera	Hand Camera
Model	RealSense D457	DS77C pro
Depth type	Stereo Depth	TOF(Time-of-flight) Depth
3D camera resolution (pixel)	640 × 480	640 × 480
3D FOV	Horizontal: 72°, Vertical: 62°	Horizontal: 70°, Vertical: 50°
Accuracy	±2%	<1%
Color camera resolution (pixel)	1280 × 800	1600 × 1200
FOV	Horizontal: 90°, Vertical: 65°	Horizontal: 77°, Vertical: 55°
Data type	Depth, IR (left + right), RGB	Depth, IR, RGB
Working distance (m)	0.32~8	0.15~5
Data interface	GigE	GigE
Size (mm), Weight (g)	124.5 × 29.5 × 36.5, 145	105 × 65 × 72.65, 581
IR Wavelength	940 nm	940 nm, 2 × 2 W VCSEL
Temperature	0~50 °C	−20~50 °C

**Table 2:** Detection accuracy of the grapes cluster of each YOLO model.

Model	valAP50	valAP95	testAP50	testAP95	Train Time Cost (s)	Predict Time Cost (ms)
YOLOv5n	0.782	0.403	0.761	0.418	2266	10.08
YOLOv5s	0.778	0.427	0.762	0.424	3848	10.15
YOLOv5m	0.766	0.442	0.770	0.454	9856	14.72
YOLOv5l	0.765	0.436	0.776	0.459	15,202	24.48
YOLOv5x	0.766	0.441	0.794	0.498	19,597	42.72
YOLOv6	0.789	0.466	0.770	0.469	3689	12.82
YOLOv7-tiny	0.758	0.378	0.769	0.400	4049	9.01
YOLOv7	0.764	0.418	0.764	0.439	4047	25.37
YOLOv7x	0.765	0.428	0.796	0.460	193,683	44.11
YOLOv8n	0.792	0.483	0.789	0.478	844	10.57
YOLOv8s	0.807	0.518	0.814	0.553	1293	10.94
YOLOv8m	0.825	0.541	0.802	0.529	6758	21.16
YOLOv8l	0.792	0.516	0.764	0.518	28,923	33.89

**Table 3:** The results of indoor experiment with different stem length.

	Stem Length				
	<20 mm	20~30 mm	30~40 mm	40~50 mm	50~60 mm
Total number	20	20	20	20	20
Correctly detected	16	16	20	19	20
Success rate (%)	80%	80%	100%	95%	100

**Table 4:** The results of outdoor experiment with real grapes.

	Stem Length				
	<20 mm	20~30 mm	30~40 mm	40~50 mm	50~60 mm
Total number	20	20	20	20	20
Correctly detected	16	16	20	19	20
Success rate (%)	80%	80%	100%	95%	100

## Data Availability

The material and data are available upon request to interested researchers.
